# Montreal Tracts on Homœopathy, No. 5

**Published:** 1888-12

**Authors:** 


MONTREAL TRACTS ON HOMŒOPATHY, No. 5. Treats of the misrepresentations of Homœopathy. Dr. Thomas Nichol is the editor.

